# Injurious Effects of the Simultaneous Administration of Calomel and Common Salt

**Published:** 1846-05

**Authors:** 


*Injurious Effects of the Simultaneous Administration of Calomel and Common Salt.*—A man affected with cerebral fever was treated by venesection and leeches. He was then ordered occasional doses of calomel, with an enema composed of a decoction of senna and a table-spoonful of common salt. The patient was in an alarming condition, but it was not anticipated that he would die speedily. Death took place, however, the same evening. The body rapidly putrefied, and presented large spots of ecchymosis. Bichloride of mercury had evidently been formed by the reaction of the two salts on each other! This fact is too important not to call for the serious attention of medical practitioners, and it imparts additional strength to the opinion, that calomel and common salt should not be given to a patient within short intervals of each other.—*Journ. de Pharm.*

*Remarks.*—We believe this *fact*, reported by our respected contemporary, to be one of those *post hoc* conclusions which are so common in therapeutics. If the calomel (in large doses) and common salt had been mixed together and thrown into the stomach, if symptoms of poisoning by corrosive sublimate had then supervened, and appearances similar to those produced by that poison had been found in the body, if, in addition to these circumstances, corrosive sublimate had been clearly detected in the stomach, there might have been good reason for attributing death to this cause, "and not to the cerebral fever." But what are the facts? Small doses of calomel enter the body by the stomach, while the common salt enters by the rectum; there was no evidence of poisoning, so far as the report goes, either during life or after death, nor was corrosive sublimate found in the body! The chemical change referred to, does take place, but only to a very minute extent, even when the substances are mixed together. The mixture requires exposure to a much higher temperature than is ever found in the stomach, to produce corrosive sublimate in perceptible quantity.—*Medical Gazette.*

